# The Prevalence of Ophthalmic Symptoms Among Adults in King Saud Bin Abdulaziz University for Health Sciences: Cross-Sectional Survey

**DOI:** 10.2196/82235

**Published:** 2026-10-14

**Authors:** Taghreed Alnahedh, Mohammed Taha, Omar Saad Alhadlaq

**Affiliations:** 1 Department of Medical Education, College of Medicine, King Saud bin Abdulaziz University for Health Sciences (KSAUHS), Prince Mutib Ibn Abdullah Ibn Abdulaziz Rd, Riyadh, Kingdom of Saudi Arabia, +966-11-4299999 Ext. 99777; 2 King Abdullah International Medical Research Center (KAIMRC), Riyadh, Kingdom of Saudi Arabia; 3 Ministry of National Guard Health Affairs, Riyadh, Kingdom of Saudi Arabia; 4 College of Medicine, King Saud bin Abdulaziz University for Health Sciences (KSAUHS), Riyadh, Kingdom of Saudi Arabia; 5 Division of Anterior Segment, Department of Ophthalmology, King Abdulaziz Medical City (KAMC-RD), Ministry of National Guard Health Affairs, Riyadh, Kingdom of Saudi Arabia

**Keywords:** eye problem, eye disease, ophthalmology, vision, visual impairment, symptoms, Saudi Arabia

## Abstract

**Background:**

Eye diseases are prevalent conditions around the world. More than 2 billion individuals globally have visual impairment. However, reducing the worldwide burden of eye disorders requires increased public awareness of prevalent eye diseases.

**Objective:**

This study aimed to investigate the prevalence of ophthalmic symptoms and associated factors among adults studying or working at King Saud bin Abdulaziz University for Health Sciences, Saudi Arabia.

**Methods:**

A cross-sectional study was conducted among adults studying or working at King Saud bin Abdulaziz University for Health Sciences, Saudi Arabia. Data were collected on ophthalmic symptoms experienced within the past 30 days, along with demographic characteristics and other relevant factors. Statistical analyses were performed to determine the prevalence of these symptoms and to identify associated factors.

**Results:**

The presence of at least 1 ophthalmic symptom was reported by 73.9% (184/249) of the respondents. The most prevalent symptoms included stinging eyes (76/249, 30.5%) and eye pain (71/249, 28.5%). Female participants reported a significantly higher prevalence of several symptoms, including stinging eyes, bloodshot eyes, swelling around the eyes, dark spots in the central field of view, foreign body sensation, and near vision difficulty. Multivariable analysis revealed that sex and the use of eye lubricants were the most significant predictors of ophthalmic symptoms (adjusted odds ratio [AOR] 3.73, 95% CI 2.02‐6.87; *P*<.001). Specifically, female participants had 3 times the odds of experiencing at least 1 ophthalmic symptom compared with male participants. Additionally, participants who used eye lubricants were 70% less likely to report ophthalmic symptoms (AOR 0.31, 95% CI 0.15‐0.6; *P*<.001).

**Conclusions:**

Ophthalmic symptoms are highly prevalent among adults in Saudi Arabia. Sex and the use of eye lubricants are key factors associated with these symptoms. These findings underscore the need for increased awareness, targeted interventions, and the promotion of eye lubricant use to reduce the burden of ophthalmic symptoms in this population.

## Introduction

One of the primary senses in humans is vision [1,2]. More than 2 billion individuals globally have visual impairment [3]. The 2 most prevalent causes of vision impairment are age-related eye disorders and refractive errors [3-5]. According to estimates from the World Health Organization (WHO), the number of people worldwide who have glaucoma will increase by 1.3 times between 2020 and 2030, from 76 million to 95.4 million, and the number of people who have age-related macular degeneration will increase by 1.2 times, from 195.6 million to 243.3 million [6].

Reducing the worldwide burden of eye disorders requires increased public awareness of prevalent eye diseases [5]. A broad range of illnesses referred to as “eye disorders and diseases” can impair vision, result in blindness, or both [6]. Early identification of eye problems is made possible by routine medical examinations, which include eye exams [7]. Patients usually see an ophthalmologist in the advanced stages of the disease, when eye discomfort and visual issues deteriorate, because the condition has a long course without symptoms [6-8]. Acute inflammatory disorders, such as chalazion and hordeolum, in contrast to chronic eye diseases, cause symptoms that require immediate attention from an ophthalmologist [9]. For a very long period, many eye conditions (including glaucoma) can have little to no symptoms [8]. Older adults may misinterpret age-related vision loss as a natural aspect of aging rather than a sign of an eye condition [10].

Several studies in Saudi Arabia reported that dry eye disease is a prevalent disorder, and studies indicate that it affects a substantial proportion of the Saudi Arabian population [11-13]. However, these studies only studied dry eye syndrome. These studies showed a high prevalence of dry eye disease in the Saudi population. Different techniques, such as online questionnaire-based surveys, have been used in these studies to evaluate the frequency of ocular symptoms, including symptoms of red eyes, dry eyes, or eyestrain. Policymakers may use information on the prevalence of eye disorders, particularly those linked to aging, and the risk factors associated with them, to develop preventative measures and eye care initiatives. The foundation for educational initiatives and tailored communication may come from the identification of variables linked to the degree of understanding regarding ocular illnesses and their risk factors. Therefore, the objective of this study was to identify the prevalence of ophthalmic symptoms and associated factors among adults studying or working at King Saud bin Abdulaziz University for Health Sciences, Saudi Arabia.

## Methods

### Study Area

The study area was King Saud bin Abdulaziz University for Health Sciences, including all campuses in Riyadh, Jeddah, and Alahsa. This manuscript was prepared in accordance with the CHERRIES (Checklist for Reporting Results of Internet E-Surveys) reporting guideline (Checklist 1).

### Study Participants

The research included various faculty members, staff, and students. Participants with ocular diseases other than nearsightedness, farsightedness, and astigmatism were excluded.

### Study Design

This study was an online questionnaire-based cross-sectional open survey.

### Study Size

The sample size was sufficient to achieve adequate statistical power and was calculated using Open-Source Epidemiologic Statistics for Public Health (OpenEpi; version 3.01) with a 95% confidence level and a 5% margin of error. A previous study showed a prevalence of 21.7% for ocular symptoms in Saudi adults [14]. As the target population was large, the default population size of 1,000,000 was retained in OpenEpi, as recommended by the software for large populations (Table 1).

- Population size (for finite population correction factor or fpc) (N): 1,000,000
- Hypothesized % frequency of outcome factor in the population (p): 21.7%±5
- Confidence limits as % of 100 (absolute ± %) (d): 5%
- Design effect (for cluster surveys-DEFF): 1

**Table 1.** Sample size for various confidence levels.

Confidence level	Sample size, n
95%	262
80%	112
90%	184
97%	320
99%	451
99.9%	736
99.99%	1029

### Sampling Technique

Participants were recruited using a convenience snowball sampling technique. The data were collected through an online questionnaire. Empirical data collection was done through a web-based anonymous questionnaire that took 10 minutes to complete. No personal identifiers were collected. As recruitment relied on university mailing lists and social media, the sample was expected to skew toward younger, more digitally engaged, and more highly educated individuals relative to the general Saudi adult population. Participation was voluntary, and no incentives were offered.

### Data Collection

A self-administered questionnaire was used for data collection (Multimedia Appendix 1). Data were collected from November 2024 to April 2025. The first part of the questionnaire comprised descriptive demographic characteristics of study participants (age, sex, marital status, job, education, income, self-reported economic status, nationality, city, and smoking status) and the second part consisted of different questions about ophthalmic symptoms and visits.

The existence of ocular symptoms was self-reported; the symptoms were not confirmed by an eye examination or medical records. However, online questionnaire-based surveys were previously frequently used to evaluate the prevalence of vision issues, primarily dry eye syndrome, and ophthalmic symptoms in various populations. Participants were asked whether they had had any of the 19 distinct ocular symptoms over the past 30 days. There were 2 possible responses: yes and no. These questions were adopted from the Ocular Surface Disease Index (OSDI) with a Cronbach α of 0.897 [15]. It should be noted that the original OSDI uses a 5-point frequency-based scale (ranging from “none of the time” to “all of the time”) to generate a continuous severity score from 0 to 100, whereas the present study used a simplified binary (yes or no) response format for each of the 19 symptom items. This represents a modified, OSDI-derived item set rather than an administration of the full validated OSDI scoring algorithm. Participants had the opportunity to review and edit their responses before final submission. All structured items within the selected survey sections were configured as mandatory responses. No randomization of survey items was used. Because technical mechanisms to prevent duplicate entries were not feasible, the dataset was manually screened for potential duplicate responses, such as identical response patterns submitted at the same timestamp.

### Data Management and Analysis Plan

Quantitative data analysis was performed using SPSS Statistics (version 27; IBM Corp). Data quality checks identified no obvious duplicate entries, and the final dataset contained no missing values. Descriptive analysis and summary statistics were presented. Categorical variables were described as frequencies and percentages. The dependent variables were the presence of ophthalmic symptoms and vision issues as reported by the participants. In addition, the models incorporated 15 independent factors, which comprised 10 sociodemographic variables, health status, and the use of contact lenses or spectacles. Subsequently, variables with a *P*<.05 in the univariable analysis were incorporated into the multivariable logistic regression model. The strength of the association between each predictor and the outcome was quantified by calculating odds ratios (ORs) and adjusted odds ratios (AORs) with 95% CIs in both models. The threshold for statistical significance in multivariable analyses was established at .05.

### Ethical Considerations

The study was conducted in accordance with the Declaration of Helsinki and approved by the institutional review board of King Abdullah International Medical Research Center (protocol NRR24/081/5; approved Oct 6, 2024). Informed consent was obtained from all participants involved in the study. Written informed consent was obtained from the participants to publish this paper. Confidentiality was maintained throughout the study period, and anonymized data were used for analysis. No incentive or compensation was offered to the participants.

## Results

### Sociodemographic Characteristics

The sociodemographic characteristics of the study participants are presented in Table 2. In the study, most participants were aged 18 to 23 years and were female. The data showed that most of the participants were faculty members and students, representing 45% (112/249) and 38.2% (95/249) of the total, respectively. In terms of education level, participants with a bachelor’s degree made up the largest proportion (75/249, 30.1%). The average monthly household income for most members exceeds SAR 10,000 (SAR 1=US $0.26 as of October 1, 2026), with 84.3% (210/249) falling into this category.

Additionally, a large portion of participants self-reported their economic status as good (137/249, 55%) or excellent (74/249, 29.7%). Furthermore, a majority of participants did not smoke or use nicotine supplements. On the basis of the data provided, over half of respondents (128/249, 51.4%) reported wearing spectacles. In contrast, only a minority (37/249, 14.9%) reported wearing contact lenses. Additionally, most respondents did not have any chronic diseases such as hypertension or type 2 diabetes mellitus. However, a significant portion of respondents had used eye lubricant in the last 2 months.

**Table 2.** Sociodemographic and lifestyle characteristics of the study participants (N=249).

Variables	Participants, n (%)
Age (y)	
18-23	81 (32.5)
24-29	37 (14.9)
30-35	27 (10.8)
36-42	54 (21.7)
43-49	28 (11.2)
≥50	22 (8.8)
Sex	
Male	88 (35.3)
Female	161 (64.7)
Current job status	
Faculty member	112 (45)
Student	95 (38.2)
Staff	42 (16.9)
Education	
High school	63 (25.3)
Bachelor’s degree	75 (30.1)
Master’s degree	42 (16.9)
Doctoral degree	69 (27.7)
Average monthly household income (SAR^a^)	
<5000	9 (3.6)
5000‐10,000	30 (12)
>10,000	210 (84.3)
Self-reported economic status	
Bad	1 (0.4)
Moderate	37 (14.9)
Good	137 (55)
Excellent	74 (29.7)
Nationality	
Saudi	206 (82.7)
Non-Saudi	43 (17.3)
City	
Riyadh	184 (73.9)
Jeddah	35 (14.1)
Alahsa	30 (12)
Do you currently smoke or use any nicotine supplements (eg, patches, gum, or e-cigarettes)?	
No	220 (88.4)
Yes	29 (11.6)
Do you wear spectacles (eyeglasses)?	
No	121 (48.6)
Yes	128 (51.4)
Which of the following chronic diseases is present?	
None	226 (90.8)
Hypertension	14 (5.6)
Type 2 diabetes mellitus	9 (3.6)
Do you wear contact lenses?	
No	212 (85.1)
Yes	37 (14.9)
Have you used eye lubricant in the last 2 months?	
No	138 (55.4)
Yes	111 (44.6)

a SAR 1=US $0.26 as of October 1, 2026.

### Prevalence of Ophthalmic Symptoms

The prevalence of ophthalmic symptoms among adults studying or working at King Saud bin Abdulaziz University for Health Sciences, Saudi Arabia, is shown in Figure 1. Around one-fifth of participants experienced symptoms such as vision deterioration, bloodshot eyes, excessive tearing, hypersensitivity to light, excessive blinking, foreign body sensation, and near vision difficulty in the last 30 days. One-third of the individuals reported stinging eyes and eye pain.

Table 3 shows the crosstabulation between ophthalmic symptoms, vision problems, and sex. We found that stinging eyes and bloodshot eyes were significantly associated with both sex. The prevalence of stinging eyes was more commonly and significantly present in female participants as compared with male participants (56/161, 35% vs 20/88, 23%; *P*=.04) in the past 30 days. More female participants reported experiencing bloodshot eyes significantly in the past 30 days than their male counterparts (44/161, 27% vs 11/88, 13%; *P*=.007). The prevalence of ophthalmic symptoms was significantly more reported by female participants than male participants, including swelling of the eyes or around eyes (18/161, 11% vs 3/88, 3%; *P*=.03), eyelid heaviness (27/161, 17% vs 2/88, 2%; *P*<.001), foreign body sensation (37/161, 23% vs 9/88, 10%; *P*=.01), and near vision difficulty (34/161, 21% vs 10/88, 11%; *P*=.05).

**Figure 1.**
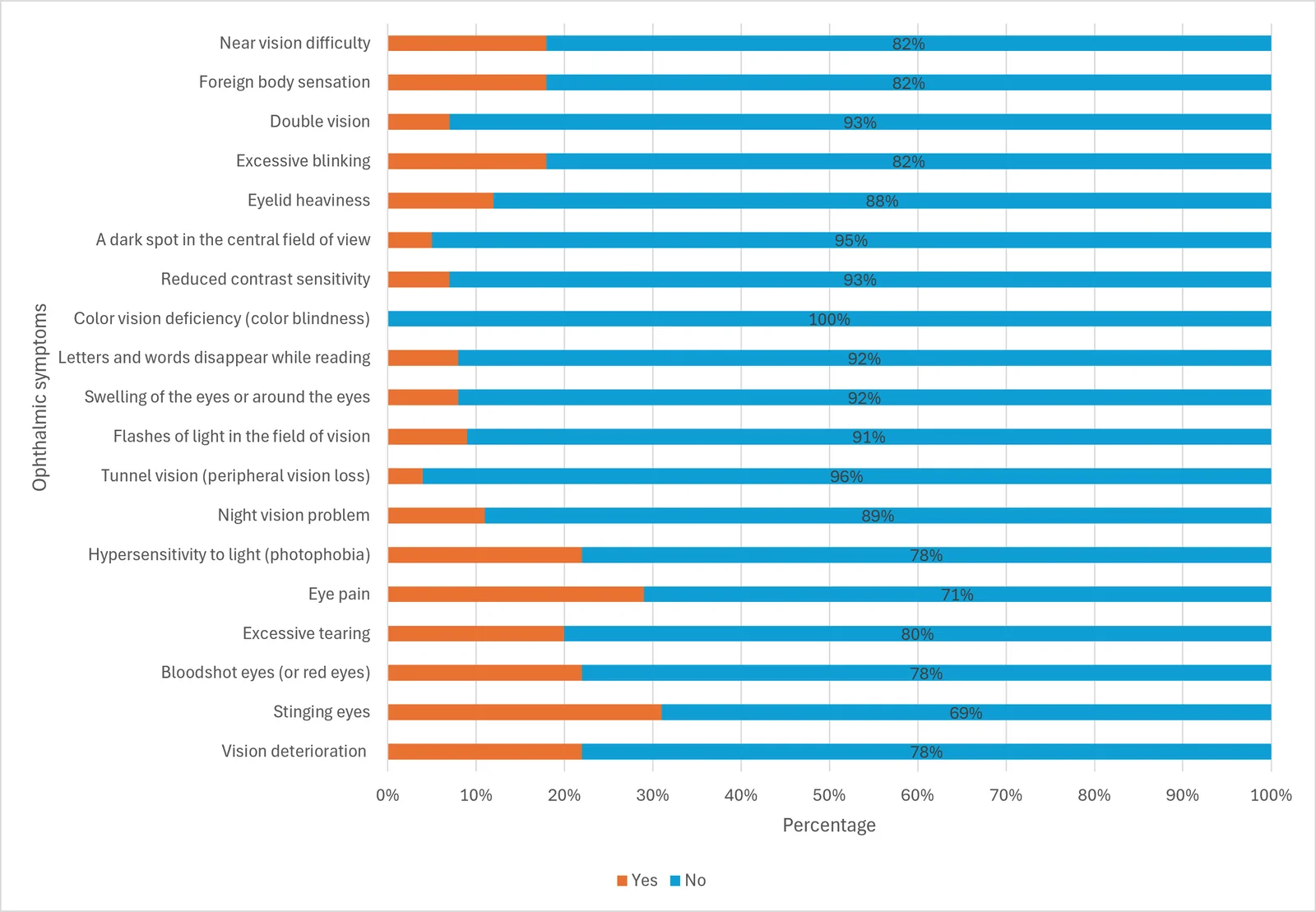
Prevalence of ophthalmic symptoms among adults in Saudi Arabia in the past 30 days (n=249).

**Table 3.** Prevalence of individual ophthalmic symptoms by sex.

Variables	Total (N=249), n (%)	Female (n=161), n (%)	Male (n=88), n (%)	*P* value
Vision deterioration	54 (21.6)	39 (24.2)	15 (17)	.18
Stinging eyes	76 (30.5)	56 (34.8)	20 (22.7)	.04
Bloodshot eyes	55 (22)	44 (27.3)	11 (12.5)	.007
Excessive tearing	49 (19.6)	32 (19.9)	17 (19.3)	.91
Eye pain	71 (28.5)	50 (31.1)	21 (23.9)	.22
Hypersensitivity to light	56 (22.5)	41 (25.5)	15 (17)	.12
Night vision problem	27 (11)	15 (9.3)	12 (13.6)	.29
Tunnel vision	10 (4)	7 (4.3)	3 (3.4)	.99
Flashes of light in the field of vision	22 (9)	15 (9.3)	7 (8)	.71
Swelling of the eyes or around eyes	21 (8)	18 (11.2)	3 (3.4)	.03
Letters and words disappear while reading	21 (8)	13 (8.1)	8 (9.1)	.78
Reduced contrast sensitivity	17 (7.2)	13 (8.1)	4 (4.5)	.29
A dark spot in the central field of view	13 (5.2)	11 (6.8)	2 (2.3)	.14
Eyelid heaviness	29 (11.6)	27 (16.8)	2 (2.3)	<.001
Excessive blinking	45 (18.1)	31 (19.3)	14 (15.9)	.51
Double vision	17 (7.2)	14 (8.7)	3 (3.4)	.11
Foreign body sensation	46 (18.4)	37 (23)	9 (10.2)	.01
Near vision difficulty	44 (17.6)	34 (21.1)	10 (11.4)	.05

### Factors Associated With the Presence of at Least 1 Ophthalmic Symptom in the Last 30 Days

Table 4 shows the factors associated with the presence of at least 1 ophthalmic symptom in the last 30 days in 249 adults in Saudi Arabia. According to univariable analysis, female participants had significantly higher odds of having 1 ophthalmic symptom than male participants (OR 1.81, 95% CI 1.03‐3.18; *P*=.03). After adjustment for potential confounders, female participants had 3 times the odds of having any 1 ophthalmic symptom than male participants in multivariable analysis (AOR 3.73, 95% CI 2.02‐6.87; *P*<.001). Study participants who used eye lubricant were 70% less likely to develop ophthalmic symptoms than those who never used eye lubricant (AOR 0.31, 95% CI 0.15‐0.6; *P*<.001).

**Table 4.** Factors associated with the presence of at least 1 ophthalmic symptom in the last 30 days (N=249).

Variables	Univariable analysis		Multivariable analysis	
	Odds ratio (95% CI)	*P* value	Adjusted odds ratio (95% CI)	*P* value
Age (y)		.81		—^a^
18-23	1 (reference)		—	
24-29	0.63 (0.19‐2.09)		—	
30-35	0.46 (0.12‐1.67)		—	
36-42	0.77 (0.18‐3.19)		—	
43-49	0.71 (0.21‐2.44)		—	
≥50	0.46 (0.12‐1.79)		—	
Sex		.03		<.001
Male	1 (reference)		1 (reference)	
Female	1.81 (1.03‐3.18)		3.73 (2.02‐6.87)	
Current job status		.73		—
Faculty member	1 (reference)		—	
Student	0.93 (0.54‐1.72)		—	
Staff	1.3 (0.55‐3.11)		—	
Education		.33		—
High school	1 (reference)		—	
Bachelor’s degree	1.1 (0.52‐2.59)		—	
Master’s degree	0.69 (0.32‐1.52)		—	
Doctoral degree	0.58 (0.25‐1.34)		—	
Average monthly household income (SAR^b^)		.42		—
<5000	1 (reference)		—	
5000‐10,000	0.4 (0.07‐2.15)		—	
>10,000	0.53 (0.19‐1.47)		—	
Nationality		.35		—
Saudi	1 (reference)		—	
Non-Saudi	0.72 (0.33‐1.47)		—	
City		.23		—
Riyadh	1 (reference)		—	
Jeddah	1.87 (0.62‐5.2)		—	
Alahsa	1.13 (0.49‐2.6)		—	
Do you currently smoke or use any nicotine supplements (eg, patches, gum, and e-cigarettes)?		.24		—
No	1 (reference)		—	
Yes	1.65 (0.71‐3.82)		—	
Do you wear spectacles (eyeglasses)?		.12		—
No	1 (reference)		—	
Yes	0.63 (0.35‐1.13)		—	
Which of the following chronic disease is present?		.65		—
None	1 (reference)		—	
Yes^c^	0.79 (0.29‐2.18)		—	
Do you wear contact lenses		.35		—
No	1 (reference)		—	
Yes	0.64 (0.29‐1.6)		—	
Have you used eye lubricant in the last 2 months?		<.001		<.001
No	1 (reference)		1 (reference)	
Yes	0.27 (0.14‐0.52)		0.31 (0.15‐0.6)	

a Not applicable.

b SAR 1=US $0.26 as of October 1, 2026.

c “Yes“ includes affirmative responses to hypertension and type 2 diabetes mellitus.

## Discussion

### Principal Findings

This study provides insights into the prevalence of ophthalmic symptoms among adults in Saudi Arabia and identifies several factors associated with these symptoms. Frequently reported symptoms include stinging eyes, bloodshot eyes, excessive tearing, and eye pain, indicating a significant burden of ocular discomfort in the population. These findings align with previous research suggesting a substantial occurrence of eye-related issues in different countries [16].

The presence of at least 1 ophthalmic symptom was found in 74% (184/249) of the respondents. A similar study conducted in Poland reported that 57% of its participants had at least 1 ophthalmic symptom in the past 30 days [5]. This discrepancy could be attributed to differences in environmental factors, lifestyle, or health care–seeking behaviors between the 2 populations. For example, the higher prevalence in Saudi Arabia may be related to environmental factors such as increased exposure to dust and sunlight. Given the high percentage of individuals experiencing at least 1 ophthalmic symptom, these findings underscore the need for comprehensive eye care services and public health initiatives focused on promoting eye health in Saudi Arabia [17].

A notable finding was the significant association between sex and certain ophthalmic symptoms. Female participants reported a significantly higher prevalence of stinging eyes and bloodshot eyes than male participants in the past 30 days. Additionally, symptoms such as swelling around the eyes, a dark spot in the central field of view, foreign body sensation, and near vision difficulty were significantly more prevalent among female participants. In fact, female participants had 3 times higher odds of experiencing at least 1 ophthalmic symptom compared with male participants in the multivariable analysis. This aligns with the epidemiology of eye disorders, as women are more susceptible to eye conditions (eg, dry eye syndrome, which is caused by hormonal changes throughout life) [5,18]. This suggests potential sex-related risk factors or reporting biases that warrant further investigation. Moreover, the higher prevalence of dry eye conditions in female participants may explain the increased number of symptomatic female participants [19,20]. A large systematic review and meta-analysis reported that around 55% of female participants have moderate to severe vision impairment [21].

The sex differences observed in the study align with existing literature that suggests hormonal factors and other physiological differences may contribute to variations in ophthalmic symptoms between male and female individuals [20,22,23]. For example, differences in tear film dynamics and meibomian gland function may contribute to a higher prevalence of certain symptoms, especially dry eye–related symptoms, among women [24]. Additionally, women might be more receptive to reporting health-related issues than men, which could contribute to the observed differences [25,26].

The study found a significant association between the use of eye lubricants and the presence of ophthalmic symptoms. Specifically, individuals who reported using eye lubricants within the past 2 months had a reduced likelihood of experiencing ophthalmic symptoms, indicating a protective effect against ocular discomfort; however, causality cannot be inferred from this cross-sectional design. Similarly, other studies reported that severely dry eyes were more common among female individuals and among contact lens users [27]. One plausible explanation for this association can be that eye lubricants provide lubrication, reduce friction, and promote corneal healing, contributing to a lower likelihood of reporting ophthalmic symptoms [28,29]. Additionally, eye lubricants may help to stabilize the tear film, minimize tear evaporation, and improve overall ocular surface health [30,31]. Therefore, regular eye lubricant use can be associated with improved ocular comfort, particularly among individuals at risk of ophthalmic symptoms [32]. Alternatively, symptomatic individuals may be more likely to initiate eye lubricant use as a self-management strategy.

Several other factors were examined for their association with ophthalmic symptoms. Other variables, such as age, education, occupation, and the presence of systemic diseases, did not show significant associations in the multivariable analysis. The study also assessed the impact of systemic diseases on the prevalence of ophthalmic symptoms. The absence of significant associations with other factors suggests that the etiology of ophthalmic symptoms may be complex and multifactorial, with individual susceptibility playing a crucial role. This highlights the potential role of individual susceptibility and underscores the need for further research to elucidate the interplay of personal, environmental, and behavioral factors in the development of ophthalmic complaints.

### Limitations

When interpreting these findings, it is important to consider the limitations of the study. First, the cross-sectional design limits the ability to establish causal relationships between risk factors and ophthalmic symptoms. Second, the reliance on self-reported data may introduce recall bias or social desirability bias. Third, this study used convenience and snowball sampling, which limits the generalizability of the findings. The resulting sample skewed toward younger, more educated individuals affiliated with a single academic health sciences institution, likely underrepresenting older adults, individuals with lower digital literacy, and those outside academic settings. Fourth, as the survey was distributed online, individuals without internet access or those less engaged with university communication channels may be systematically underrepresented, potentially inflating or deflating true symptom prevalence estimates.

### Conclusions

In conclusion, this study provides valuable insights into the prevalence and determinants of ophthalmic symptoms among adults at King Saud bin Abdulaziz University for Health Sciences, Saudi Arabia. The findings highlight the importance of sex and eye lubricant use as key factors associated with these symptoms. Further research is needed to confirm these findings and to develop effective strategies for preventing and managing ophthalmic symptoms in this population. These findings have several important implications for public health and clinical practice. The high prevalence of ophthalmic symptoms underscores the need to raise awareness among the public and health care professionals about these issues. Given the sex differences observed, targeted interventions may be necessary to address the specific needs of female and male individuals. Encouraging the use of eye lubricants, particularly among individuals at risk, may help reduce the burden of ophthalmic symptoms. Additional research is needed to explore the underlying causes of ophthalmic symptoms and to identify effective strategies for prevention and management.

## Supplementary material

10.2196/82235 Multimedia Appendix 1 Ophthalmic symptoms questionnaire.

10.2196/82235 Multimedia Appendix 2 Underlying dataset used in the study.

10.2196/82235 Checklist 1 Checklist for Reporting Results of Internet E-Surveys (CHERRIES).

## Abbreviations

AOR: adjusted odds ratio
CHERRIES: Checklist for Reporting Results of Internet E-Surveys
OR: odds ratio
OSDI: Ocular Surface Disease Index
WHO: World Health Organization
