# Supplementary material for: The Prevalence of Ophthalmic Symptoms Among Adults in King Saud Bin Abdulaziz University for Health Sciences: Cross-Sectional Survey
Source: Online J Public Health Inform. 2026 Oct 14;18:e82235. doi: 10.2196/82235 (PMC13664986; doi:10.2196/82235)
Supplement: Supplementary file 1 [file ojphi-v18-e82235-s001.docx]

**The knowledge and prevalence of ocular symptoms through an online questionnaire-based survey in King Saud bin Abdulaziz University for Health Sciences**

Questionnaire

|  | | | - | | |
| --- | --- | --- | --- | --- | --- |
| a. Age | 18 to 23 years 24 to 29 years  30 to 35 years 36 to 42 years  43 to 49 years 50 years and above | | | | |
| b. Marital Status | | 1. Married ☐  2. Single ☐  3. Divorced ☐ | | c. Gender | Male = 1 ☐  Female = 2 ☐ |
| d. Current Job status | | 1. faculty member 2. students  3. Staff  4. Other______ | | | |
| e. Education | | 1. Primary ☐ 2. High School ☐ 3. Diploma ☐ 4. Bachelor's degrees ☐ 5. Master's degree ☐ 6. PhD degree ☐ 7. Other _______ | | | |
| f. Average Monthly Household Income | | Income, SAR *  <5000 ☐ 5000–10,000 ☐ >10,000 ☐ | | | |
| g. Self-reported economic status | | 1. Good 2. Moderate 3. bad | | | |
| h. Nationality | | 1. Saudi national 2. Non-Saudi national | | | |
| i. City | | 1. Riyadh 2. Jeddah 3. Alahsa | | | |
| j. Do you currently smoke or use any nicotine supplements (e.g., patches, gum, e-cigarettes)? | | 1. Yes 2. No | | | |

1. Do you wear spectacles (eyeglasses)?

a. Yes
b. No

2. Presence of chronic disease

a.	Yes

b.	No

3. If your answer is yes for question no. 2, which of the following chronic disease is present?

a. Type 2 diabetes

b. Hypertension

4.  Do you wear contact lenses?

a.	Yes

b.	No

5. Do you use eye lubricant

a.	Yes

b.	No

**Ocular symptoms**

| **17. In the last 30 days, have you had any of the eye symptoms listed below? (indicate all that apply)** | | |
| --- | --- | --- |
|  | **Yes** | **No** |
| Vision deterioration |  |  |
| Stinging eyes |  |  |
| Bloodshot eyes (or red eyes) |  |  |
| Excessive tearing |  |  |
| Eye pain |  |  |
| Hypersensitivity to light (photophobia) |  |  |
| Night vision problem |  |  |
| Tunnel vision (peripheral vision loss) |  |  |
| Flashes of light in the field of vision |  |  |
| Swelling of the eyes or around the eyes |  |  |
| Letters and words disappear while reading |  |  |
| Color vision deficiency (color blindness) |  |  |
| Reduced contrast sensitivity |  |  |
| A dark spot in the central field of view |  |  |
| eyelid heaviness |  |  |
| excessive blinking |  |  |
| double vision |  |  |
| foreign body sensation |  |  |
| near vision difficulty |  |  |
